# Non-invasive imaging of engineered human tumors in the living chicken embryo

**DOI:** 10.1038/s41598-017-04572-1

**Published:** 2017-07-10

**Authors:** Benedict Jefferies, Florian Lenze, Anuja Sathe, Nguyen Truong, Martina Anton, Rüdiger von Eisenhart-Rothe, Roman Nawroth, Philipp Mayer-Kuckuk

**Affiliations:** 1 Department of Orthopedics, Technical University Munich, Munich, Germany; 2 Department of Urology, Technical University Munich, Munich, Germany; 3 Departments of Molecular Immunology and Experimental Oncology, Klinikum rechts der Isar, Technical University Munich, Munich, Germany

## Abstract

The growing interest in engineered tumor models prompted us to devise a method for the non-invasive assessment of such models. Here, we report on bioluminescence imaging (BLI) for the assessment of engineered tumor models in the fertilized chicken egg, i.e, chick chorioallantoic membrane (CAM) assay. One prostate cancer (PC-3) and two osteosarcoma (MG63 and HOS) cell lines were modified with luciferase reporter genes. To create engineered tumors, these cell lines were seeded either onto basement membrane extract (BME) or gelfoam scaffolds, and subsequently grafted *in vivo* onto the CAM. BLI enabled non-invasive, specific detection of the engineered tumors on the CAM in the living chicken embryo. Further, BLI permitted daily, quantitative monitoring of the engineered tumors over the course of up to 7 days. Data showed that an extracellular matrix (ECM) composed of BME supported growth of reporter gene marked PC-3 tumors but did not support MG63 or HOS tumor growth. However, MG63 tumors engineered on the collagen-based gelfoam ECM showed a temporal proliferation burst in MG63 tumors. Together, the data demonstrated imaging of engineered human cancer models in living chicken embryos. The combination of CAM assay and BLI holds significant potential for the examination of a broad range of engineered tumor models.

## Introduction

Engineered tumor models are attractive for cancer research because they enable controlled replication of the human tumor stroma, that is the ECM and non-cancerous cells within the ECM, and are believed to offer new insights into tumor biology including therapy response^1–4^. Often based on advances in tissue engineering, examples of engineered tumor models include tumor organoids and tumor cells on porous artificial or tissue-derived scaffolds^5, 6^. Engineered tumor models can be studied *in vitro* or transplanted *in vivo*. Hosts for *in vivo* studies include rodents^7^, the mainstay in preclinical oncology models. In this study, however, we explore the fertilized chicken egg as *in vivo* host for engineered tumors.

The fertilized chicken egg harbors the CAM, a vascularized extraembryonic membrane, and between day 1 and 16 of chick embryonic development the CAM can be utilized to assay *in vivo* biology^8^. A unique set of features make the CAM assay an attractive *in vivo* model. For example, it offers (1) low costs, (2) no need for ethics approval, (3) a naturally immunodeficient model, and (4) direct physical access to the assay site^9^. The CAM assay is particularly well established for angiogenesis research^10^, which led to its use in studies examining tumor angiogenesis and dissemination^11^. Exploring engineered tumor models in the CAM assay, however, poses a challenge because the tumor cells reside and proliferate within a scaffold, limiting the value of visual or manual inspection of tumor progression. To circumvent this challenge, we reasoned that BLI is suited for non-invasive and quantitative engineered tumors in CAM assays. This imaging technique has emerged as a powerful and frequently used mean to monitor cells non-invasively *in vivo* ^12, 13^. It is based on the expression of a luciferase reporter protein in cells^14^. Upon administration of the luciferase substrate luciferin, the luciferase-expressing cells emit light, and hence become detectable using optical detection systems^15^. Several key advantages have contributed to the success of BLI particularly using the Firefly luciferase reporter in pre-clinical animal models. These advantages include (1) no toxicity and excellent biodistribution of the substrate D-luciferin, (2) extremely low background and good light penetration, enabling detection of bioluminescence in virtually all mouse tissues, (3) the low costs and comparably simple instrumentation of BLI, and (4) the straight-forward, user friendly application of the technique^16, 17^. Taking advantage of these characteristics, the aim of this study was to develop and validate BLI of engineered tumors in the living chicken embryo.

## Results

### Prostate cancer cell line PC-3-GL exhibited bioluminescence and fluorescence properties

**Figure 1:**
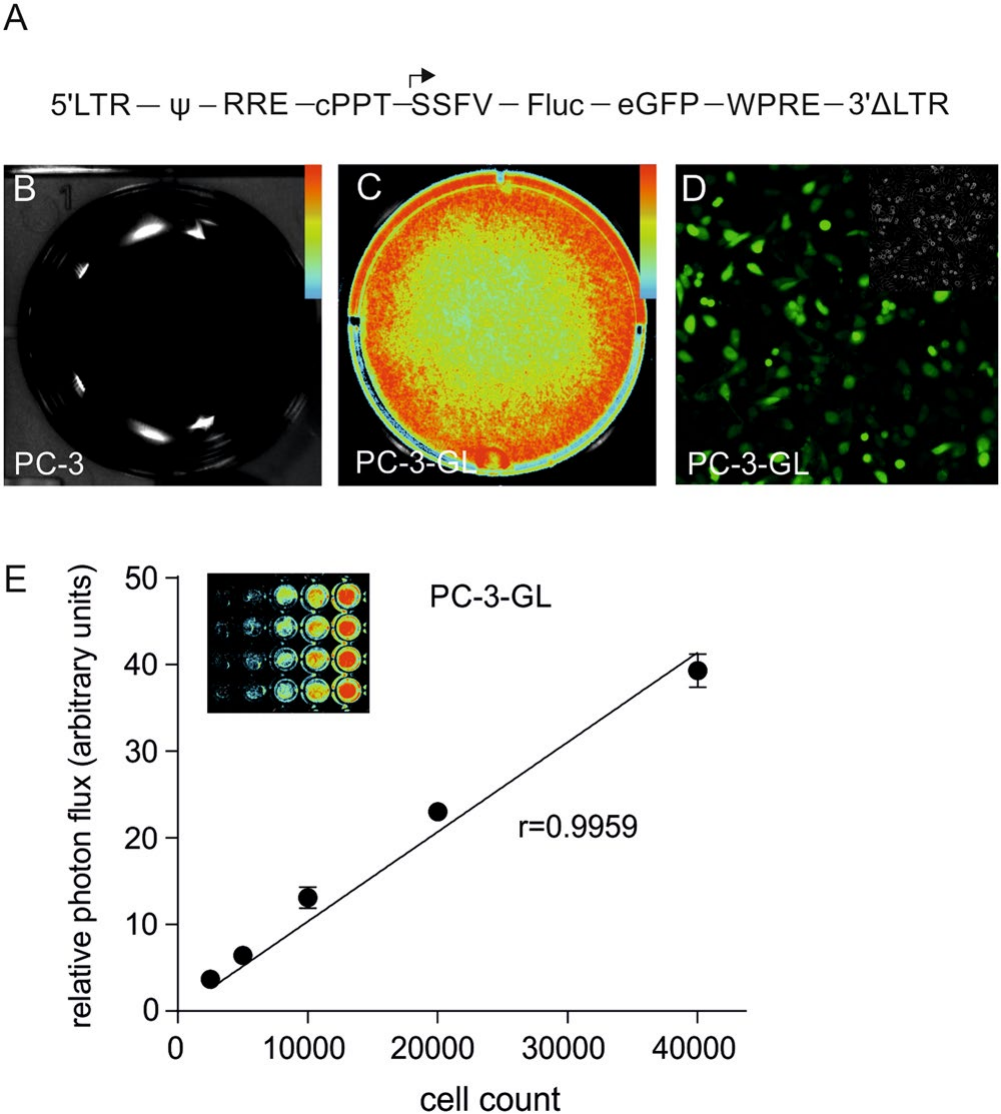
Validation of the reporter gene modified PC-3-GL cell line. (**A**) Schematic presentation of the bicistronic lentiviral vector. (**B**) *In vitro* BLI of PC-3 control cells. (**C**) *In vitro* BLI of PC-3-GL cells. (**D**) Fluorescence microscopy on PC-3-GL cells. The insert shows the corresponding brightfield image. (**E**) Correlation of bioluminescent light production and PC3-GL cell number. All color bar inserts range from 0 (blue) to 16383 (red) units photon flux. Abbreviations: Ψ, psi packaging sequence; RRE, rev response element; cPPT, central polypurine tract element; SSFV, spleen focus-forming virus promoter; eGFP, enhanced green fluorescent protein; Fluc, firefly luciferase; WPRE, woodchuck hepatitis virus post‐transcriptional regulatory element.

To initially test the proposed imaging method, we chose to examine the human prostate cancer cells PC-3, because PC-3 cells have previously been investigated in CAM assays^18^. PC-3 cells were lentivirally transduced with an enhanced green fluorescent protein/Firefly luciferase reporter gene (Fig. 1A). The transduced cell line was termed PC-3-GL. As expected, visualization of luciferase-mediated bioluminescence showed no signal in the parental PC-3 cells (Fig. 1B), while a signal was obtained from the transduced PC-3-GL cells (Fig. 1C). A corresponding fluorescence signal from the green fluorescent protein was also readily detected (Fig. 1D). Further, titration experiments showed an excellent linear relationship between light production and PC-3-GL cell number (Fig. 1E). Thus, BLI could be used to monitor cell growth in PC-3-GL cells.

### Bioluminescence imaging of engineered PC-3-GL-BME tumors upon engraftment on the CAM of fertilized eggs

**Figure 2:**
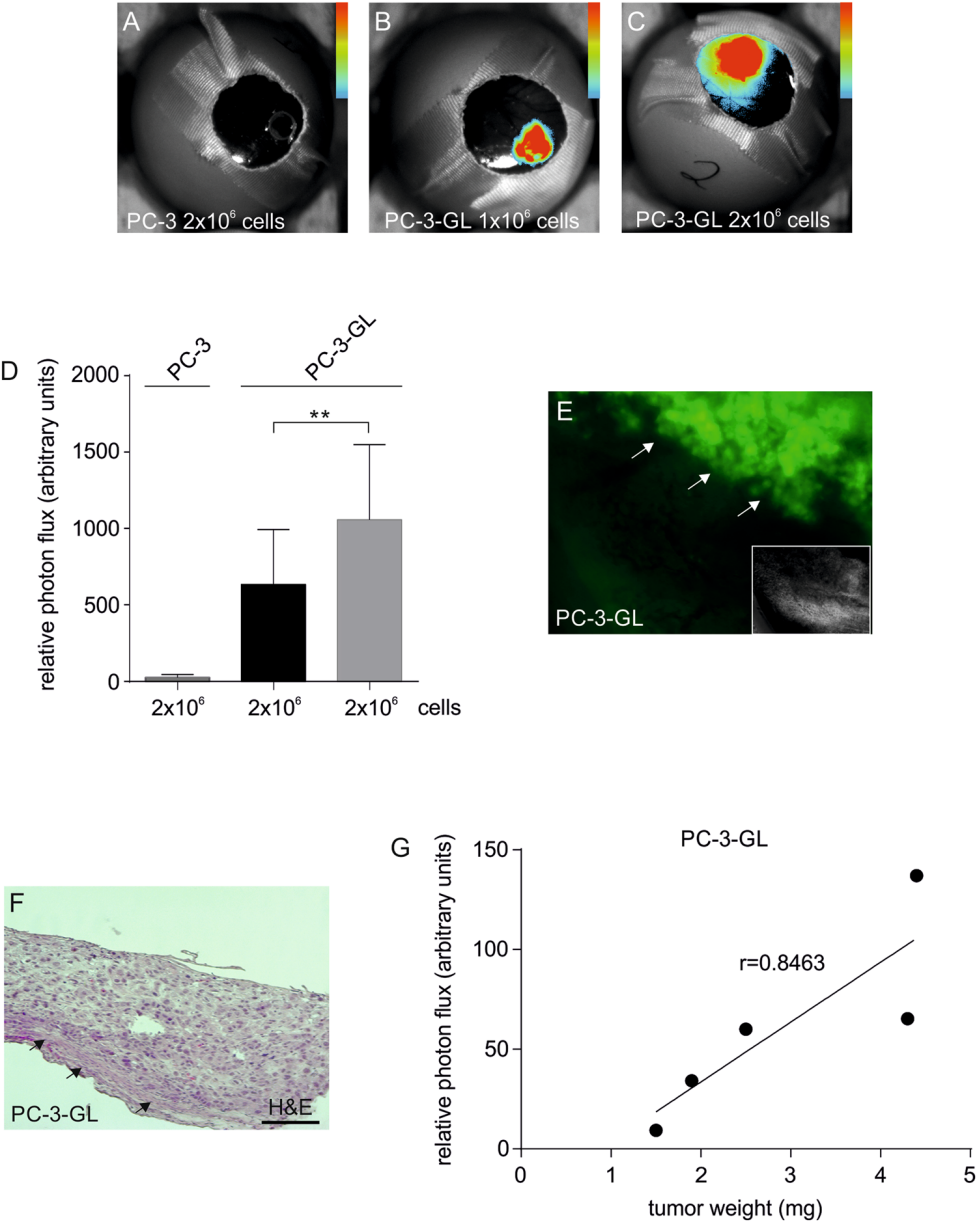
BLI of engineered prostate cancer-BME scaffolds in the living chick embryo. (**A**) *In vivo* egg BLI of a PC-3-BME control scaffold. (**B** and **C**) Representative images of *in vivo* BLI of PC3-GLBME scaffolds seeded with (**B**) 1 × 10^6^ (**C**) and 2 × 10^6^ cells. (**D**) Quantitative BLI analysis are plotted as means with standard deviation (n = 4). (**E**) Post-mortem fluorescence microscopy on a PC-3-GL-BME scaffold and adjacent CAM tissue. Small insert: Corresponding PC-3-BME control scaffold. White arrows delineate the engineered tumors. (**F**) Fixed, paraffin-embedded section of the engineered tumor with adjacent CAM stained with hematoxylin and eosin. (**G**) Plot of *in vivo* measured BLI signal against postmortem measured weight of the engineered tumor. All color bar inserts range from 0 (blue) to 16383 (red) units photon flux. ** < 0.01.

For *in vivo* experiments, tumors composed of 2 × 10^6^ parental PC-3 cells as well as 1 × 10^6^ or 2 × 10^6^ PC-3-GL cells were engineered using basal membrane extract (BME) as a scaffold. Engineered tumors were grafted onto CAMs in fertilized chicken eggs and BLI performed on day 10 of embryonic development. Imaging revealed no signal from 2 × 10^6^ cells wild type controls (Fig. 2A). In contrast, when analyzing PC-3-GL cells a signal was detected and correlated to the number of cells seeded onto the CAM (Fig. 2B and C). Quantitation showed an approximately 1.7-fold difference in *in vivo* BLI signal intensity between 1 × 10^6^ cells and 2 × 10^6^ cells (Fig. 2D). Post-mortem, the CAM with the engineered tumor was removed for *in situ* fluorescence microscopy. Images showed expression of GFP in the tumor tissue (Fig. 2E). Subsequent histology confirmed presence of tumor tissue in the engineered scaffolds (Fig. 2F). Lastly, the correlation between bioluminescence signal *in vivo* and tumor weight measured post-mortem was found to show a very good correlation (Fig. 2G). Of note, tumors smaller than 1 mg in weight could not be correlated reliably to BLI signal (not shown).

### Osteosarcoma cell lines MG63-RL and HOS-RL showed bioluminescence and fluorescence properties

**Figure 3:**
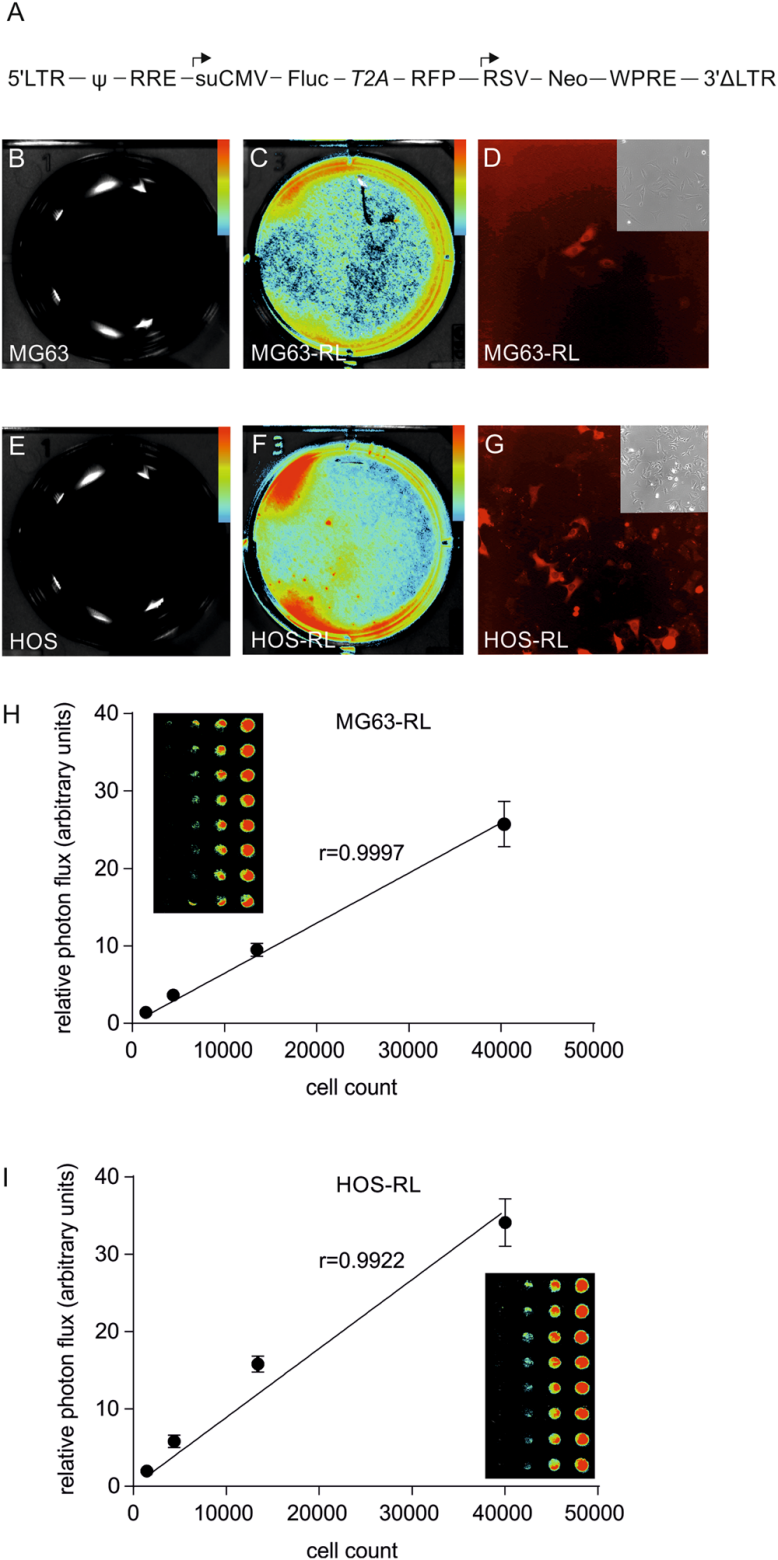
Validation of the reporter gene modified MG63-RL and HOS-RL cell lines. (**A**) Schematic presentation of the bicistronic lentiviral vector. (**B**) *In vitro* BLI of MG63 control cells. (**C**) *In vitro* BLI of MG63-RL cells. (**D**) Fluorescence microscopy on MG63-RL cells. The insert shows the corresponding brightfield image. (**E**) *In vitro* BLI of HOS control cells. (**F**) *In vitro* BLI of HOS-RL cells. (**G**) Fluorescence microscopy on HOS-RL cells. The insert shows the corresponding brightfield image. (**H**) Correlation of Bioluminescent light production and MG63-RL cell number. (**I**) Correlation of bioluminescent light production and HOS-R cell number. All color bar inserts range from 0 (blue) to 16383 (red) units photon flux. Abbreviations: Ψ, psi packaging sequence; RRE, rev response element; suCMV, CMV cytomegalovirus promoter; Fluc, firefly luciferase; T2A, thoseaasigna virus 2A self-cleavage peptide; RFP, red fluorescent protein; RFP; RSV, rouse sarcoma virus promoter; Neo, neomycin locus; WPRE, woodchuck hepatitis virus post‐transcriptional regulatory element.

The human osteosarcoma cell lines MG63 and HOS were transduced with a Firefly luciferase/red fluorescent protein reporter gene (Fig. 3A), because there have been concerns about GFP expression in osteosarcoma cells^19^. The transduced cell lines were termed MG63-RL and HOS-RL. While parental MG63 and HOS control cells showed no detectable bioluminescence (Fig. 3B and E), MG63-RL and HOS-RL cells demonstrated a very robust signal (Fig. 3C and F). A corresponding fluorescence signal from the RFP was readily detected (Fig. 3D and G). Subsequent titration experiments showed a linear relationship between the level of light emission and MG63-RL or HOS-RL cell number (Fig. 3H and I). Thus, BLI could be used to monitor cell growth.

### Bioluminescence imaging visualized engineered MG63-RL-BME and HOS-RL-BME tumors upon engraftment on the CAM of fertilized eggs

**Figure 4:**
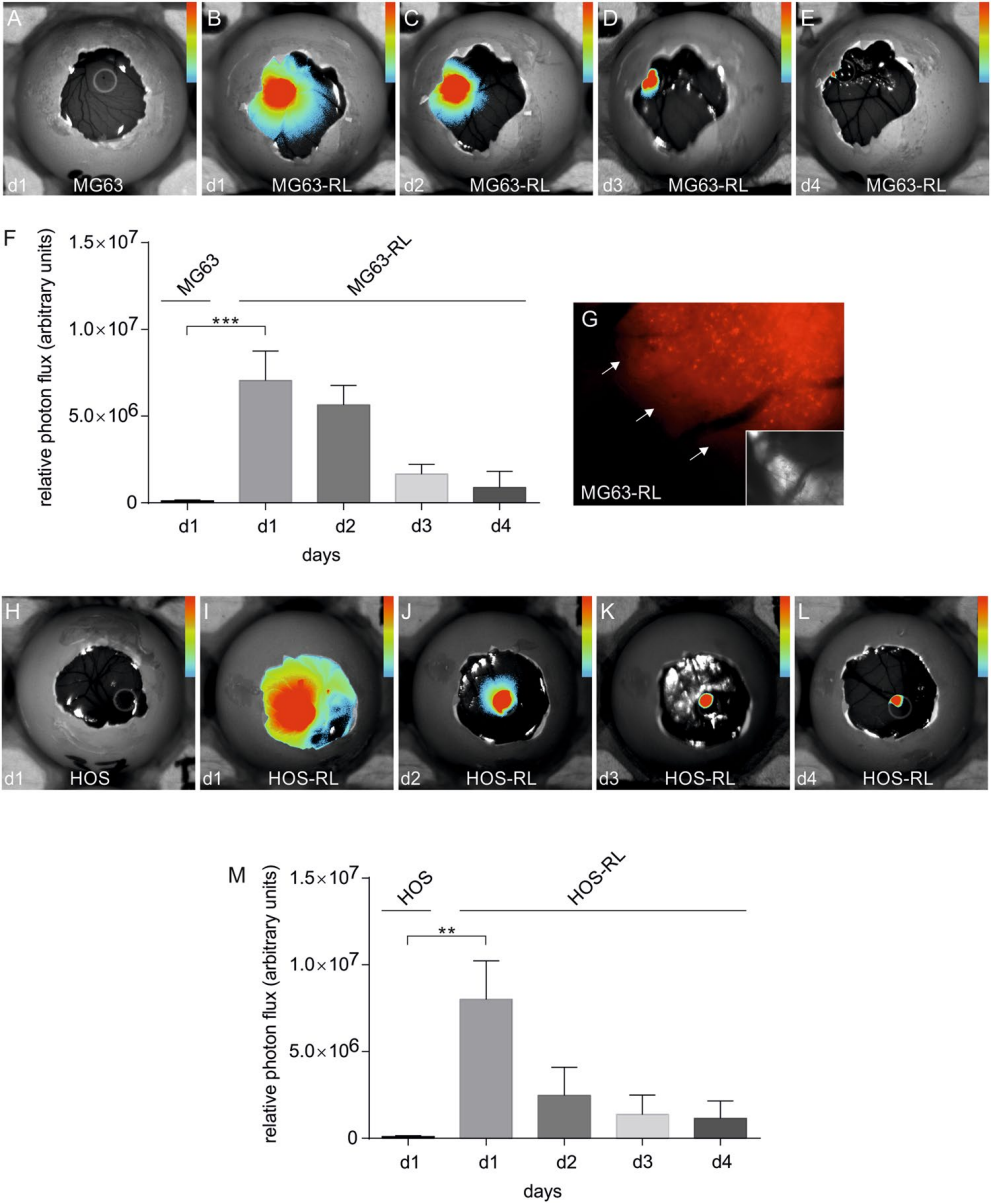
BLI of engineered osteosarcoma-BME scaffolds in the living chick embryo. (**A**) *In vivo* egg BLI of a MG63-BME control scaffold. (**B**–**E**) Representative images of daily *in vivo* egg BLI of MG63-RL-BME scaffolds over a course of 4 days. (**F**) *In vivo* egg BLI of a HOS-BME control scaffold. (**G**–**J**) Representative images of daily *in vivo* egg BLI of HOS-RL-BME scaffolds over a course of 4 days. (**K**) Quantitative BLI analysis are plotted as means with standard deviation (n = 4). Large insert: Post-mortem fluorescence microscopy on MG63-R-BME scaffold and adjacent CAM tissue. Small insert: Corresponding MG63-BME control scaffold. The inserts show the matching brightfield images. White arrows delineate the engineered tumors. (**L**) Corresponding quantitative BLI analysis are plotted as means with standard deviation (n = 3). All color bar inserts range from 0 (blue) to 16383 (red) units photon flux. **p < 0.01, ***p < 0.001.

Engineered osteosarcoma tumors were composed of BME and MG63-RL or MG63 parental control cells. No signal was detected from latter tumors (Fig. 4A). In contrast, a strong BLI signal was seen the BME-MG63-GL tumors and enabled daily imaging of individual eggs (Fig. 4B–E). The BLI was not associated with any noticeable toxicity. Signal quantitation demonstrated an 87-fold increased signal from BME-MG63-GL tumors as compared to BME-MG63 parental tumors (Fig. 4F). The engineered BME-MG63-RL tumors showed a 20%, 76% and 87% decrease in signal over the course of 3 days (Fig. 4F). Despite the drop in BLI signal, post-mortem *in situ* microscopy on day 4 showed of the CAM harboring BME-MG63-RL tumors showed red fluorescence from the BME-MG63-RL tumor (Fig. 4G), but not from the corresponding MG63-BME control tumor (Fig. 4G). Hence, confirming the origin of the BLI signal. To test if the above findings were specific to MG63 cells, we further investigated HOS cells. Observations made in engineered BME-HOS tumors mirrored the findings in engineered MG63 tumors. A specific BLI signal was detected from the BME-HOS-GL tumors (Fig. 4H and I) and was found to decrease by 69%, 83% and 86% over 4 days (Fig. 4H–K and L).

### Bioluminescence imaging recorded growth of engineered MG63-RL-gelfoam tumors upon engraftment on the CAM of fertilized eggs

**Figure 5:**
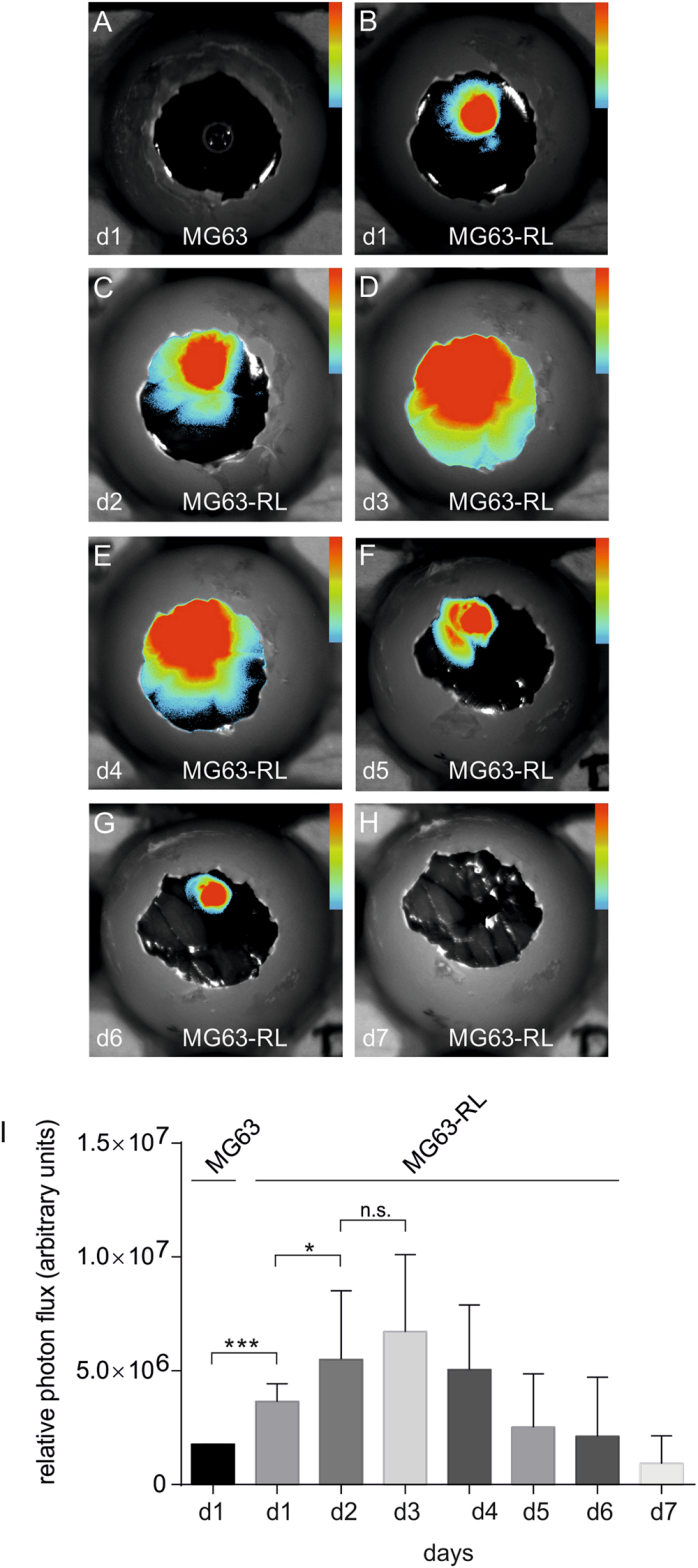
BLI of engineered osteosarcoma-gelfoam scaffolds in the living chick embryo. (**A**) *In vivo* egg BLI of MG63-gelfoam control scaffolds. (**B**–**H**) Representative images of daily *in vivo* egg BLI of MG63-R-gelfoam scaffolds over a course of 7 days. (**I**) Corresponding quantitative BLI analysis expressed as means with standard deviation for the group n = 13. All color bar inserts range from 0 (blue) to 16383 (red) units photon flux. *p < 0.05, ***p < 0.001.

Next, we tested whether a gelfoam scaffold supported MG63-RL tumor cells differently than BME. Similar to the experiments above, BLI was used to assess the MG63-RL-gelfoam tumor on the CAM. In contrast to the BME environment, MG63-RL-gelfoam tumors demonstrated a significant increase in BLI signal from d1 to d2 and remained statistically not altered for additional two days. On d4, a statistically insignificant 25% drop in BLI signal was observed as compared to the maximum signal on d3 (Fig. 5I). By d5 the BLI signal decreased to 63% (p < 0.01) compared to d3, and on d6, and d7 a further signal decrease of 61% and 86%, respectively, was observed (Fig. 5I). In comparison, on the BME scaffold signal had already dropped by 87% on d4 (Fig. 4F).

## Discussion

In this method paper, we described the development of a CAM assay for non-invasive, live imaging of engineered human tumor models *in vivo*. We were not aware of any detailed report on BLI applications in a CAM assay prior to our study. Our study was motivated by the limitations of traditional *in vivo* measures of tumor growth on the CAM which typically rely on visual inspection and light microscopy. These means are semi-quantitative and more importantly have limited value for engineered tumor models, because the tumor mass in these models forms with a scaffold. Here, we show that these limitations can be overcome by the use of non-invasive BLI. The presented method will integrate seamlessly with standard modes of observation in the egg, such as visual inspection through the opening of the egg or post-mortem application such as the use of polymerase chain reaction for the detection of Alu sequences specific for human cancer xenografts. A limitation of BLI, however, is that it does not provide single cell resolution and it is important that it can also be combined with fluorescence-based methods. In this regard, previous work has employed fluorescence labeling using a green fluorescent protein (GFP) and demonstrated that this approach enables detection *in situ* ^20^, while more recent work has shown intravital microscopy of GFP-tagged cells in the fertilized chicken egg^21, 22^.

We suggest several advantages of the presented imaging. First, the combination of CAM assay and BLI is cost effective and has potential for high through-put, due to the lack of housing costs and minimal space requirements. Including the egg purchase but excluding delivery, we calculated the running costs to image one egg daily over a period of 8 days to about 4 US dollars. Second, the presented imaging strategy could capitalize on the large portfolio of existing luciferase reporters for multi-modal imaging of various aspects of cancer biology. Third, we speculate that the BLI imaging in the fertilized egg is compatible with other imaging modalities including magnetic resonance imaging. Fourth, the presented imaging should not interfere with the classical angiogenesis read-out of CAM assays, therefore permitting combined tumor and angiogenesis measure in a non-invasive fashion. Lastly, the unrestricted physical access to the CAM, and thus ease of administration of biological or pharmacological agents makes the assay attractive for pre-clinical cancer drug testing. In this respect, we have conducted preliminary experiments on MG63-RL seeded gelfoam scaffolds, indicating that daily administration of growth factors, i.e. platelet-derived growth factor BB, may increase cell survival and growth beyond day 4 (BJ and PMK personal communication). With respect to our experimental approach, we detected the bioluminescent light emission with a Fusion FX7 standard 4.2 × 10^6^ pixel charge-coupled device (CCD) camera system, and thus our detection sensitivity is likely to be inferior to a systems using a high-end, cooled CCD camera. In consequence, we expect that use of latter imaging equipment will increase sensitivity and/or decrease imaging times. Third, in our study we took advantage of the unrestricted tissue penetration of D-luciferin and the physical access to the CAM to administer the D-luciferin directly onto the engineered tumor. Other studies have shown the possibility to inject systemically into the circulatory system of the fertilized egg^23, 24^, and it is likely that this route of administration can also be explored for D-luciferin administration. However, due to the technical challenges of administration into the circulatory system in this model, survival rates of the eggs are optimized when using a topical application.

Imaging data in our study was derived from engineered human prostate and osteosarcoma tumors. To initially develop the presented method, we used PC-3 prostate cells. These cells have been previously studied in conventional CAM assays^18^, and were more recently reexamined in CAM assays with respect to metastases formation^25, 26^. With regard to latter, we speculate that the imaging method presented here has additional value of the whole body assessments of metastases formation in developing chicken ex ovo. As our future research interests are geared towards osteosarcoma, we subsequently engineered human osteosarcoma tumor models. We found that osteosarcoma cells engrafted in BME on the CAM do not readily proliferate upon engraftment, but rather vanish. The uncompromised expression of the red fluorescent protein in our post-mortem analysis strongly indicates that cell death rather than reporter gene silencing caused the decline in bioluminescence signal. Our observations are consistent with work by Balke *et al*.^27^, whose findings indicated that the capacity of MG63 and HOS cells to form solid tumors on the CAM is modest. However, we speculate that models of suppressed growth have value in the examination of cell death or even dormancy. Latter is supported by work showing that BME induces dormancy features in human breast and murine osteosarcoma cancer cells^28^. In contrast to the growth behavior in BME, osteosarcoma cells seeded on gelfoam, a denatured collagen ECM, proliferated at least over a period of three days. Although type I collagen is the major component of bone, we note that the tumor model we used is very simple and it will be important to extend our work using for example the demineralized, decellularized bone scaffold reported to provide a tumor niche for Ewing sarcoma^29^. Altogether, our data supports the further development of bioluminescent CAM assays for *in vivo* cancer research.

## Methods

### Chemicals and reagents

All chemicals were from Sigma (St. Louis, MO). The two lentiviral constructs used to direct constitutive reporter gene expression under control of a spleen focus-forming virus or cytomegalovirus have been both reported before^30, 31^. Matrigel BME was purchased from BD Bioscience (Billerica, MA). D-luciferin was obtained from Zellbio (Ulm, Germany). Gelfaom was from Pfizer (New York, NY).

### Cell culture

Human prostate cancer PC-3 and human osteosarcoma MG63 cell lines were obtained from the American Type Culture Collection (Manassas, VA). The human HOS cell line was a kind gift from the Nathrath lab (Helmholtz Center Munich, Germany). Prostate cancer cells were grown in RPMI-1640 medium containing 10% fetal bovine serum (FBS) and 100 U/ml penicillin/100 μg/ml streptomycin, while osteosarcoma cells were cultured in Dulbecco’s modified Eagle’s medium (DMEM), supplemented with 10% FBS, 100 U/ml penicillin/100 μg/ml streptomycin, and 1% L-glutamine. All cell culture media and supplements were purchased from Gibco (Waltham, MA). Cells were maintained at 37 °C in a humidified 5% carbon dioxide atmosphere, and sub-cultured at a 1:5 ratio.

### Reporter gene transduction

Lentiviral reporter gene transductions followed established methods^32^. Briefly, cells were seeded in 0.5 ml medium at 50,000 cells/well in 24 well plates and cultured for 16 hrs. For transduction, cells were exposed to ~1 × 10^7^ IFU/mL and then incubated for 72 hrs. Transduced cells were sorted for fluorescent protein expression using a FACSVantage (BD Bioscience). Cells modified with a neomycin resistance gene, were selected in the presence of 500 µg/ml G418 (Geneticin, Gibco) prior to FACS. All reporter gene expressing cell lines were subsequently cultured as described above for the parental cells.

### CAM Assay

Fertilized chicken eggs were purchased from a commercial chicken farm (Brueterei Sued, Regenstauf, Germany), and couriered to the laboratory. Experiments were performed in accordance with relevant guidelines and regulations, and approved under a waiver by the Technical University Munich. After arrival, eggs were maintained at 37 °C, and for the first 4 days incubated with the smaller convexity pointing downwards. Prior to opening on chick embryonic development day 4, eggs were turned so that the smaller convexity pointed upwards. All subsequent procedures were carried out under aseptic conditions. Materials used to manipulate the CAM were sterilized. Eggs were opened mechanically on top creating an approximately 10 mm opening, carefully minimizing any contamination of the interior with shell fragments. The opening was sealed with parafilm and eggs incubated for an additional 4 days. On embryonic development day 8, the opening was enlarged to about 20 mm. The following day, tumor cells were seeded. First, the CAM was lacerated gently using a lens paper, as reported previously^33^, to generate a micro-hemorrhage and thus facilitate engraftment. Next, a silicon seeding ring with 5 mm and 4 mm outer and inner diameter, respectively, was placed on the site of this hemorrhage. For tumor cell seeding onto BME, 2 × 10^6^ cells were re-suspended in 10 µl of standard medium, then 10 µl BME was added and the cells carefully seeded onto the CAM utilizing the seeding ring. For cell seeding onto gelfoam, 2 × 10^6^ cells were resuspended in 20 µl medium and applied to a gelfoam cube of approximately 2 mm size. Subsequently, the cube was engrafted on to the CAM utilizing the seeding ring. Eggs were subjected to *in vivo* BLI as described below. For sacrifice, eggs were exposed to dry ice for the duration of one hour. Subsequently, the CAM was excised for *in vitro* BLI and fluorescence imaging on embryonic development day 13.

### Bioluminescence imaging (BLI)

Bioluminescence imaging was performed in Fusion FX7 imager (Peqlab, VWR, Darmstadt. Germany). For *in vitro* BLI, 1.5 × 10^6^ parental or transduced cells were seeded in 6-well-plates. For titration experiments, transduced cells were seeded as 3-fold dilutions in 96-well-plates. Imaging was conducted sixteen hours after seeding at a 10 min. exposure time for both experiments after adding D-luciferin luciferin (ZellBio GmbH, Ulm, Germany) to a final concentration of 150 μg/ml. For *in vivo* BLI, eggs were imaged in sets of two controls and two experimental eggs. The first day of imaging (d1) corresponded to day 10 of embryonic development. Subsequently, the eggs were imaged for bioluminescent signal on a daily basis. Prior to image acquisition, 30 µl of a 30 mg/ml D-luciferin solution in phosphate buffered saline were administered directly onto the CAM. A brightfield image was acquired, followed by bioluminescence acquisition for 10 min. Brightfield and bioluminescence images were then merged. All quantitation was performed using ImageJ.

### Fluorescence imaging

For microscopy a Zeiss Axio Observer Z1 microscope (Carl Zeiss, Oberkochen, Germany) equipped with an HBO 100 illumination source and an AxioCam MRc detector were used. Red fluorescent protein expression was detected at an excitation and emission wavelength of 545 nm and 620 nm, respectively. Fluorescence microscopy was carried out *in vitro* in standard tissue culture flasks. In addition, excised CAMs were examined for fluorescence on the final day of imaging. Image contrast on the fluorescence images was adjusted in Corel Draw.

### Tumor weighing

Harvested tumors were trimmed under a Zeiss Stemi DV4 stereomicroscope ensuring that surrounding CAM was removed. Tumor weight was measured using a fine balance after transferring trimmed tumors to pre-weighed micro-centrifuge tubes containing 200 µl PBS.

### Histological staining

Tissue samples were fixed for in 4% paraformaldehyde for 16 h at 4 °C. Xylene and a descending ethyl alcohol series were used for deparaffinization and rehydration of 2.5 µM sections from formalin-fixed, paraffin-embedded tumor tissue. Next, tissues were stained with Mayer’s hematoxylin solution (Hospital Pharmacy, Klinikum rechts der Isar, Munich, Germany) for 5 min., followed by successive washes in tap water, deionized water and 95% acid ethanol for 5 min., 2 min. and 20 sec., respectively. Then sections were stained in alcoholic Eosin B (Hospital Pharmacy) for 2 min., followed by dehydration with an ascending alcohol series and mounting with Entellan NEW (Merck Chemicals GmbH, Darmstadt, Germany). Slides were examined using a Zeiss Axio Vert.A1 microscope and images were obtained using Zen Lite 2012 software (Carl Zeiss).

### Statistics

Statistical comparisons were made using unpaired or paired t-tests. A p value of less than 0.05 was considered significant.
